# Association of food consumption patterns and nutritional status of children under 5 years from rural households in Northern regions, Namibia

**DOI:** 10.1186/s40795-024-00833-1

**Published:** 2024-03-18

**Authors:** Maria Angula, Anthony Ishola, Muvari Tjiurutue, Nozizwe Chigonga, Michael Sulyok, Rudolf Krska, Chibundu N. Ezekiel, Jane Misihairabgwi

**Affiliations:** 1 Department of Human, Biological, and Translational Medical Sciences, School of Medicine, University of Namibia, Windhoek, Namibia; 2 Department of Pharmaceutical Sciences, School of Pharmacy, Faculty of Health Sciences and Veterinary Medicine, University of Namibia, Windhoek, Namibia; 3 Department of Biochemistry, Microbiology and Biotechnology, School of Science, University of Namibia, Windhoek, Namibia; 4 Independent Nutrition Consultant, Windhoek, Namibia; 5 Department of Agrobiotechnology (IFA-Tulln), Institute of Bioanalytics and Agro-Metabolomics, University of Natural Resources and Life Sciences, Vienna, Konrad Lorenz Str. 20, Tulln, A-3430 Austria; 6 Institute for Global Food Security, School of Biological Sciences, Queen’s University Belfast, University Road, Belfast BT7 1NN, Northern Ireland, United Kingdom

**Keywords:** Child health, Food consumption patterns, Malnutrition, Nutritional status

## Abstract

**Background:**

Many developing countries, Namibia included, have a high prevalence of malnutrition among children, especially in rural subsistence farming areas where inadequate food supply is common. Poor diets in children under 5 years may result in negative health impacts. This study determined the association of food consumption patterns and nutritional status of children under 5 years from rural households in Oshana and Oshikoto regions in Namibia.

**Method:**

Employing a cross-sectional descriptive design, 377 children under 5 years participated in this study using purposive sampling. Validated dietary diversity and food frequency questionnaires were used to obtain information on demographic characteristics, commonly consumed food per week, and meal frequencies for the recruited children. Anthropometric measurements were obtained to assess nutritional status of children using Emergency Nutrition Assessment (ENA) software. Descriptive and inferential statistics were computed using the IBM® SPSS® Statistics (Statistical Package for Social Sciences) version 27.

**Results:**

Staple foods, mostly grains, roots and tubers, along with flesh foods, legumes and nuts were commonly consumed. Vitamin A-rich fruits and vegetables were solely consumed in Oshana region (10.7%) and not in Oshikoto. Oshana exhibited a lower dietary diversity score (4±1 SD) compared to Oshikoto (5±1 SD). The prevalence of adequate feeding practices varied, with Oshana having 38.8% meeting minimum milk feeding frequency (MMFF), 55.6% minimum dietary diversity (MDD), 69.8% minimum meal frequency (MMF), and 27% minimum acceptable diet (MAD). In Oshikoto, these figures were lower at 2%, 7%, 32%, and 0.5%, respectively. Stunting, underweight, wasting, and overweight were also documented, with slight differences between the two regions. The study did not find association between nutritional status and MMFF, MDD and MAD. However, significant associations were found between specific food types, amount of food, breastfeeding length, MMF and malnutrition indicators in both regions (*p<*0.05).

**Conclusion:**

Most study participants consumed locally available staple foods. Stunting, underweight, and wasting were prevalent among children in the two regions which were significantly associated to the amount of food consumed, MMF and/ some food types. Improving food environments and eliminating access barriers to diversified diets can mitigate high prevalence of malnutrition among rural children.

## Introduction

Poor nutrition is an important public health concern, affecting many children globally, mostly in developing countries. It is responsible for most mortality and morbidity affecting millions of children worldwide [1, 2]. Globally, Sub-Saharan Africa is reportedly responsible for one-third of all malnourished children with about 39% stunted, 10% wasted and 25% underweight for children under the age of 5 [3]. In Namibia, malnutrition remains a concern, with the prevalence of stunting in children reported to be about 24%, above the 20% cut-off point stipulated by the World Health Organisation (WHO) [4]. Oshana and Oshikoto regions are among the most densely populated regions in the Northern part of Namibia, with a large proportion of the population living in rural areas and depending on subsistence farming for livelihood [5]. Rural communities in Namibia often face challenges such as inadequate food supply, which can contribute to malnutrition. According to the report of Chotard [6], the levels of nutritional status indicators, such as stunting, wasting, and underweight in Northern regions, were reported nearly twice as high as those in the central regions of Namibia. Thus, understanding the food consumption patterns and nutritional status of children in these regions can help identify specific needs and inform targeted interventions.

The consumption pattern of healthy food in early childhood can contribute to optimum growth and development of a child and may lead to a long, healthy life [7]. Poor diets in children under 5 years contribute to developmental disabilities, increased risk of illnesses and deaths in childhood, and may have negative health impacts into adulthood [3, 8]. The prevalence of under-nutrition, which includes wasting, stunting and underweight among children reflects poor nutritional practices [9]. This indicates that children need to consume a diversified group of foods to meet their nutrient demands for physical and mental growth. Knowing the food consumption patterns of children can aid health care providers to provide targeted guidance to childcare providers on the facilitation of the development of healthy eating habits. Also, it will help to improve food systems and the overall food environment in childcare centres or family childcare homes that provide significant portions of daily food intakes [10].

Minimum dietary diversity, minimum meal frequency and minimum acceptable diet are key indicators of proper feeding practices [11, 12]. As per WHO guidelines, non-breastfeeding children should receive a minimum of five out of the seven essential food groups while, breastfeeding children should receive a minimum of four food groups in addition to breastmilk. These include grains, roots, and tubers; dairy products; legumes and nuts; flesh foods such as meat, fish, poultry, and organ meats; vitamin-A rich fruits and vegetables; eggs, and other fruits and vegetables. These variety is essential to maintain normal growth and development [11, 12]. However, reports have indicated a concerning trend as globally, less than a quarter of the children under 5 years, primarily from Sub-Sahara Africa, are not receiving an adequate minimum dietary diversity, minimum meal frequency and minimum acceptable diet [12–14].

The diets of children under age 5 in rural areas are usually staples such as cereal foods with minimal intake of fruits and vegetables, and this may contribute to malnutrition because children at this crucial stage of growth require sufficient nutrients to meet their growth demands. Despite this being the case, limited studies have been conducted to investigate the association between food consumption patterns and nutritional status of children from the rural households in Namibia. Therefore, this study sought to assess the food consumption patterns and nutritional status of children under 5 years of age and link their nutritional status to food consumption patterns. There is a need of interventions to improve the health and nutritional status of children, to minimise mortality and morbidity among children.

## Methodology

### Study design and population

A cross-sectional descriptive study was conducted in rural households of Oshana and Oshikoto regions. A total of 377 (Oshana = 178; Oshikoto = 199) non-breastfeeding children under the age of 5 were purposively selected and recruited to participate in this study. Purposive sampling was employed to specifically target the study participants based on their age group and exclusively from the two regions, ensuring representation from the diverse demographic groups. Findings may not be generalizable to children under 5 years in other regions of Namibia or the entire country. The number of children included in the study was calculated based on a formula previously described by Naing et al. [15] as: N = [Z^2^ P (1 - P)] / d^2^ whereby; N is the required population size, Z is the confidence level of 95% (1.96 standard value), P is the proportion of children under 5 years from the two regions, according to the earlier Namibia population and housing census of 2011 data [16] and d is the precision at 5%. Therefore, this study recruited 377 children under five years from Oshana and Oshikoto regions.

The recruitment involved the inclusion of at least one child per family depending on the number of children within the required age in a family. The parents or children’s care providers gave written consent for their children to participate in the study and the children participated willingly. Childcare providers were interviewed to answer questions on household demographic and child food consumption patterns.

### Demographic characteristics

Validated dietary diversity questionnaires and food frequency questionnaires were used to obtain information about the age, gender, feeding practices, and health status of the children. Information of the occupation and educational level attained by each child’s parent/childcare provider were also collated.

### Food consumption pattern

The validated dietary diversity questionnaires and food frequency questionnaires contained aspects relating to food frequency and consumption patterns. Thus, the information about commonly consumed foods in the past 7 days, the number of days the child consumed the food, the frequency of consumption per day, and the amount of food consumed were retrieved for each child. The commonly consumed foods were then grouped into the following 7 main food groups:

- grains, roots, and tubers
- dairy products
- legumes and nuts
- flesh foods (meat, fish, poultry, and organ meats)
- vitamin A-rich fruits and vegetables, eggs
- other fruits and vegetables.

Each childcare provider of the target child answered the interview questions.

### Anthropometric data

Anthropometric data were collected to determine the prevalence of nutritional status indices (underweight, wasting and stunting) in children under 5 years. Measurements taken for each child were weight, middle-upper arm circumference (MUAC) and height. Children were weighed in light clothing and bare footed on a calibrated portable electronic digital scale in kilograms (kg) to the nearest 0.1kg. To ensure accuracy, measurements were taken twice for weight and a third measurement was taken if they differed with more than 0.2 kg and the average weight (kg) measurement was used. The MUAC measurements were taken using the MUAC tape to the nearest millimetres (mm). Height was measured to the nearest centimetre (cm) using a United Nations International Children's Emergency Fund (UNICEF) stadiometer. Height measurements were taken twice, and a third time if the two initial measurements differed by more than 0.3 cm.

### Data analysis

All the data was analysed on IBM® SPSS® Statistics version 27. Data interpretation was achieved using descriptive statistics and presented in frequencies and percentages. Anthropometric data was deposited into ENA software to calculate child height-for-age z-scores (HAZ), weight-for-age z-scores (WAZ), weight-for-height z-scores (WHZ) and MUAC cut-offs using the WHO growth standards reference data [17]. A child with a z-score less than minus 2 standard deviations (-2SD) for HAZ was considered stunted, whereas a child with a z-score below -2SD for WAZ were considered underweight. Those with WHZ less than -2SD were considered wasted. Children whose WHZ were greater than 2SD above the mean were considered overweight. Children were considered severely affected if their z-scores were below -3SD For HAZ, WAZ and WHZ. Children with MUAC cut-off values greater or equal to 115 and less than 125mm were considered moderately wasted while those with MUAC cut-off values less than 115mm were considered severely wasted (severe acute malnutrition).

In assessing food consumption patterns, a child who received food from five or more food group for a minimum of five days in the past 7 days met the criteria for minimum dietary diversity, while those who received for less than five days were classified as not receiving the minimum dietary diversity. Minimum dietary diversity score was calculated using the following formula below:

$$\frac{\mathrm{Number}\,\mathrm{of}\,\mathrm{under}\,5\,\mathrm{years}\,\mathrm{children}\,\mathrm{who}\,\mathrm{received}\,\mathrm{food}\,\mathrm{from}\,5\,\mathrm{or}\,\mathrm{more}\,\mathrm{food}\,\mathrm{groups}\,\mathrm{in}\,\mathrm{the}\,\mathrm{past}\,7\,\mathrm{days}}{\mathrm{Number}\,\mathrm{of}\,\mathrm{under}\,\mathrm{five}\,\mathrm{years}\,\mathrm{children}}\,\mathrm X100$$

Regarding meal frequency, a child who consumed solid, semi-solid, or soft foods four times or more per day for five or more days in the past 7 days met the minimum meal frequency. Meeting the minimum milk feeding frequency required a child to receive two milk feedings per day for five or more days in the past 7 days. Children who achieved at least the minimum dietary diversity (excluding dairy products) from four out of six food groups, along with meeting the minimum meal frequency and milk feeding frequency, were considered to have met the minimum acceptable diet, adapted from WHO / UNICEF [12].

The dietary diversity, minimum acceptable diet and minimum meal frequency indicators were coded as 0 and 1, representing children who did not meet and who met the criteria for minimum dietary diversity, minimum acceptable diet, and the minimum meal frequency, respectively. Cross-tabulations using Chi-square determined if there was an association between independent variables including food consumed, minimum dietary diversity, minimum meal frequency, minimum acceptable diet and the dependent variables (nutritional status) of children. The results were deemed significant at *p* values <0.05. Bonferroni correction controlled the Type I error by adjusting significance levels. Significant results had *p* values ≤ adjusted significance level. Multinomial logistic regression measured the association between food consumption patterns and nutritional status using odds ratios (ORs). OR > 1 indicated increased odds of the outcome while OR < 1 indicated decreased odds of the outcome, with significance at *p* < 0.05.

## Results

### Demographic characteristics of rural households

The data in Table 1 presents the demographic data of the study participants. The mean age in months of children in this study was higher in the Oshikoto region (37 ± 13.6) compared to Oshana region (33 ± 13.06). Among the children who participated in the study from Oshana region, more than half (56%) were females while 44% were males, while for Oshikoto region, 51% were females and 49% were male.

**Table 1:** Gender presentation of children under 5 years who participated in study (*n* = 377)

**Region**	**Gender**	**Frequency (*n*=377)**	**Percentage (%)**
Oshikoto	Male	98	49
	Female	101	51
Oshana	Male	79	44
	Female	99	56
	Total	377	100

**Fig. 1:**
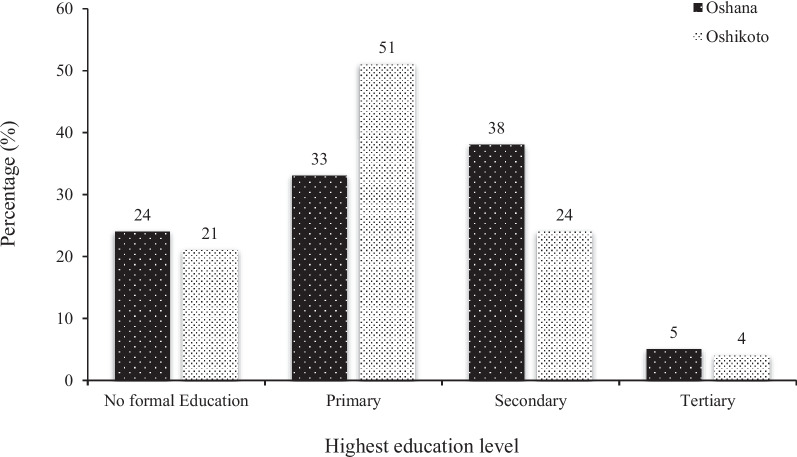
Education level of childcare providers from Oshana and Oshikoto regions

Regarding the education level of the childcare providers, less than a quarter of these women from both regions received informal education while no more than 5% of the women in each region received tertiary education. The majority (38% and 51%) of these caregivers received secondary and primary levels of education in Oshana and Oshikoto regions, respectively (Fig. 1).

**Fig. 2:**
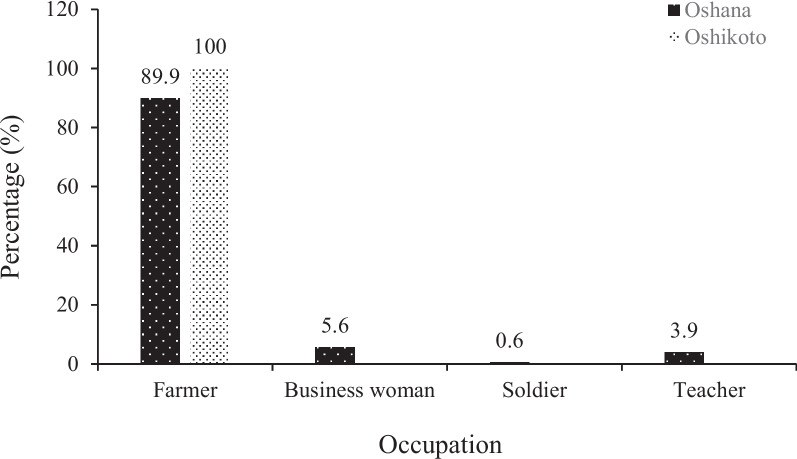
Occupation of childcare providers from Oshana and Oshikoto regions

A higher proportion of the childcare providers from the two regions (Oshana: 90%; Oshikoto: 100%) were subsistence farmers compared to other occupations (Fig. 2). About 10% of childcare providers from Oshana region were businesswomen, teachers, or soldiers, whereas none of these professions were recorded in the Oshikoto region.

**Fig. 3:**
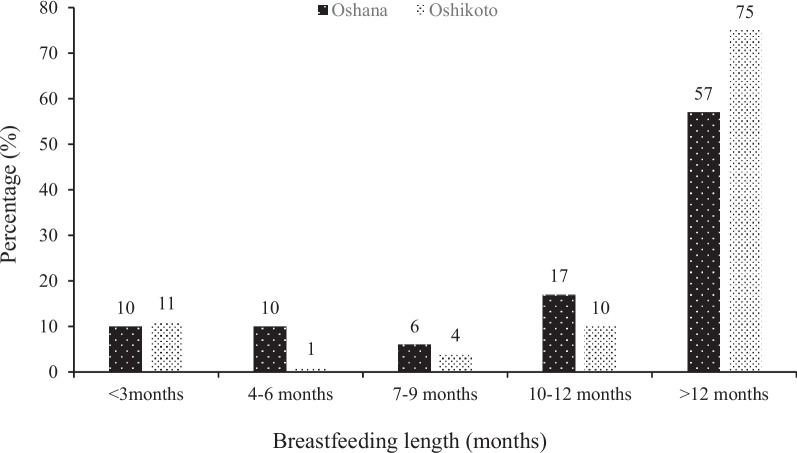
Breastfeeding length of children under 5 years from Oshana and Oshikoto regions

With respect to breastfeeding of the children, 57% and 75% of the children from Oshana and Oshikoto regions, respectively, were breastfed for more than 12 months (Fig. 3). About 20% and 12% of the children in Oshana and Oshikoto regions, respectively, were breastfed for six months and less. Concerning the children’s health challenges, majority of the childcare providers indicated that 96% and 99% of children from Oshana and Oshikoto regions, respectively, did not experience any health challenge.

### Commonly consumed food among children under 5 years

**Fig. 4:**
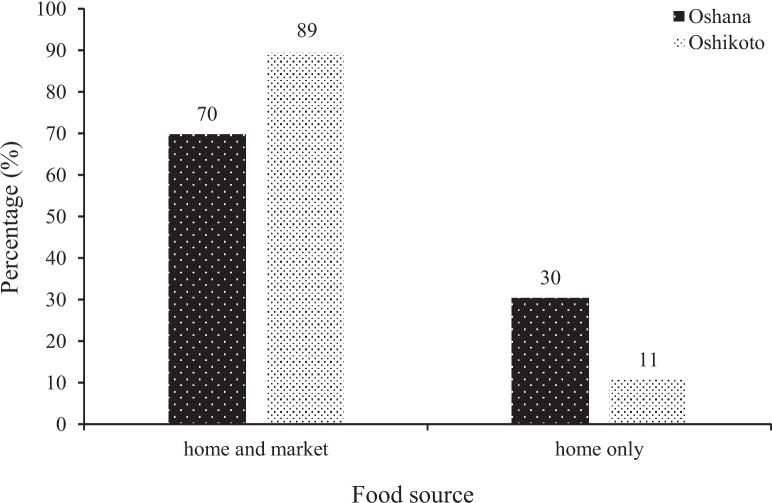
Source of food fed to children under 5 years from Oshana and Oshikoto regions

The sources of foods fed to the children are presented in Fig. 4. Children in the two regions were fed with foods sourced at home and from markets. However, a higher proportion (at least 70%) of children in the regions consumed foods sourced from both home and markets. Only 11% and 30% of the children from Oshana and Oshikoto regions, respectively depended exclusively on food produced at home.

**Fig. 5:**
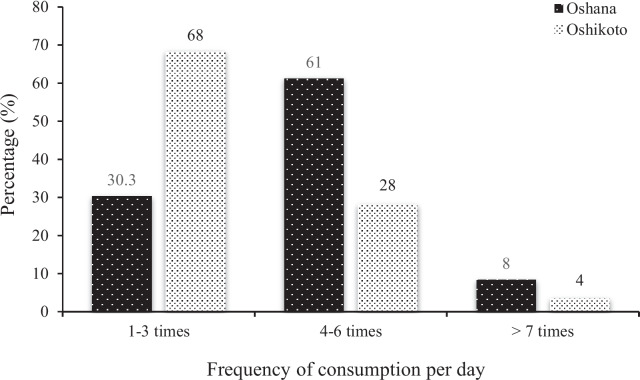
Frequency of meal consumption among children under 5 years

The frequency of meal consumption per day (solid, semi-solid, or soft foods) by the children in the two regions varied from 1–3 times to >7 times per day (Fig. 5). In the Oshana region, the highest frequency of meal consumption per day was 4–6 times recorded for 61% of the children, while in the Oshikoto region the highest daily meal consumption frequency of 68% was recorded for 1–3 times. Less than 10% of the children in each of the regions had daily meal consumption exceeding 7 times.

The data on minimum meal frequency among children from the Oshana and Oshikoto regions is presented in Table 2. According to the results, less than half of the children from Oshikoto region (32%) met the recommended minimum meal frequency while, in the Oshana region, approximately 70% (69.7%) of the children met the recommended minimum meal frequency.

**Table 2:** Minimum meal frequency among children from Oshana and Oshikoto regions.

Minimum meal frequency	Oshana	Oshikoto
Met minimum meal frequency	124 (69.7)	33 (31.7)
Did not meet minimum meal frequency	54 (30.3)	136 (68.3)

**Fig. 6:**
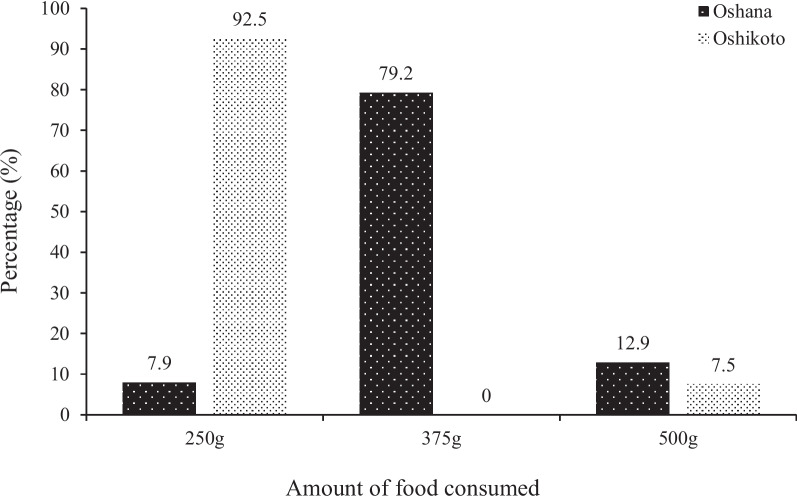
Amount of food consumed per meal by children under 5 years from Oshana and Oshikoto regions

Regarding the quantity of meals consumed (Fig. 6), 79% of the children from Oshana region consumed 375g of food per meal while only 8% and 13% of the children in the same region consumed 250g and 500g of food, respectively. A higher proportion (92.5%) of children from the Oshikoto region consumed 250g of food as a meal while 8% consumed 500g of food per meal.

The children under 5 years from Oshana and Oshikoto regions consumed food from 5 and 6 food groups, respectively, in a week (Table 3). The most popular food groups consumed by the under 5 years children from the Oshana region, in order of magnitude, included grains, roots, and tubers (100%), followed by flesh foods (100%), then legumes and nuts (98.3%), other fruits and vegetables (96.1%), and dairy products (55.1%). Only a small percentage of children consumed Vitamin A rich fruits and vegetables (10.7%), and none of the children consumed eggs. Similarly, the primary food groups consumed by the children from the Oshikoto region in a week included grains, roots, and tubers (99%), flesh foods (98.5%), legumes and nuts (96%), and other fruits and vegetables (78.9%). A small proportion of the population consumed dairy products (8.5%). Children in the Oshikoto region did not consume fruits and vegetables rich in vitamin A, nor did they consume eggs.

**Table 3:** Main food groups consumed by children under 5 years in Oshana and Oshikoto regions in a week

Food item	1-3 times per day		4-5 times a day		6-7 times a day		Never	
Grains, root and tubers	Oshana	Oshikoto	Oshana	Oshikoto	Oshana	Oshikoto	Oshana	Oshikoto
					178 (100)	195 (98)		4 (2)
Vitamin A rich fruits and vegetables	19 (10.7)						159 (89.3)	199 (100)
Other fruits and vegetables	44 (24.7)	70 (35.2)	131 (73.6)	85 (42.7)		2 (1)	3 (1.7)	
Flesh foods		147(73.9)		43 (21.6)				9 (4.5)
Dairy products	29 (16.3)		54 (30.3)		15 (8.4)		80 (44.9)	
Eggs							178 (100)	199 (100)
Legumes and nuts		84(42.2)	32 (18)	103 (51.8)	145 (81.5)	3 (1.5)	1 (0.6)	

The variety of individual food items consumed by children in these two regions were diverse, with 13 and 16 different foods recorded in Oshikoto and Oshana, respectively (Table 4). In the Oshana region, the food types consumed within the grains, roots and tubers food group included traditional cereal opaque beverage (*oshikundu*) (100%), pearl millet thick/thin porridge (94%), maize thick/thin porridge (42%), rice (53%) and macaroni (39.3%). For flesh foods children consumed chicken (98%), beef (96%) and fresh fish (95%). Legumes and nuts primarily consisted of beans (98%) while other fruits and vegetables encompassed traditional leafy vegetables (*ombidi*) (96%) and watermelons (0.6%). Dairy products included milk (55%) while Vitamin A rich fruits and vegetables (10.7%) included mangoes, pumpkins, and papaya.

**Table 4:** Types of foods commonly consumed by children under 5 years from Oshana and Oshikoto regions

Food item	Frequency (%)	
	Oshana (*n*=178)	Oshikoto (*n*=199)
Pearl millet thick/ thin porridge	167 (93.8)	193 (97)
Maize thick/ thin porridge	74 (41.6)	164 (82.4)
*Oshikundu*	178 (100)	195 (98)
Beans	175 (98.3)	191 (96)
*Ombidi*	171 (96.1)	157 (78.9)
Fresh fish	169 (94.9)	177 (88.9)
Chicken	174 (97.8)	195 (98)
Beef	170 (95.5)	170 (85.4)
Dried fish	159 (89.3)	152 (76.4)
Rice	95 (53.4)	171 (85.9)
Macaroni	70 (39.3)	103 (51.8)
Milk	98 (55.1)	17 (8.5)
Vitamin A fruits and vegetables	19 (10.7)	-
Potatoes	-	9 (5.1)
Watermelons	1 (0.6)	–
Snacks	3 (1.7)	–

In the Oshikoto region, the types of food consumed by the under 5 years children in a week within the grains, roots and tubers food group included the traditional cereal opaque beverage (*oshikundu*) (98%), pearl millet thick/thin porridge (97%), rice (86%), maize thick/thin porridge (82%), macaroni (52%) and potatoes (5.1%). Flesh foods included chicken (98%), fresh fish (89%) and beef (85%). Legumes and nuts consumed by the children primarily consisted of beans (96%) while, other fruits and vegetables mainly comprised traditional leafy vegetables (*ombidi*) (79%). Dairy products included milk (8.5%).

Table 5 shows individual dietary diversity scores of under 5 years children from Oshana and Oshikoto regions. The average dietary diversity score for the Oshikoto region was 4±1 SD, whereas for the Oshana region, it was 5±1 SD. In the Oshana region, 55.6% of the children achieved the minimum dietary diversity, while less than half (44.4%) had inadequate dietary diversity. Conversely, in the Oshikoto region, only 7% of the children attained the minimum dietary diversity, while a substantial majority (93%) had inadequate dietary diversity (Table 6).

**Table 5:** Individual dietary diversity scores for the under 5 years children from Oshana and Oshikoto regions

Diversity scores	Oshana	Oshikoto
1	-	-
2	2 (1.1)	4 (2)
3	11 (6.2)	43 (21.6)
4	66 (37.1)	138 (69.3)
5	87 (48.9)	14 (7)
6	12 (6.7)	-
7	-	-

**Table 6:** Prevalence of feeding indicators among children under 5 years from Oshana and Oshikoto regions

Indicator	Oshana	Oshikoto
Minimum dietary diversity		
Met MDD	99 (55.6)	14 (7)
Inadequate dietary diversity	79 (44.4)	185 (93)
Milk feeding frequency		
Met MMFF	69 (38.8)	4 (2)
Inadequate milk feeding frequency	109 (61.2)	195 (98)
Minimum acceptable diet		
Inadequate MAD	130(73)	198 (99.5)
Met MAD	48 (27)	1 (0.5)

In relation to milk feeding frequency (Table 6), less than half (38.8%) of the children from Oshana region met the required milk feeding frequency, while only 2% of the children from Oshikoto region received the required milk feeding frequency. Consequently, among the children from Oshana region 27% received the minimum acceptable diet while from Oshikoto region, only 0.5% of the children met the minimum acceptable diet.

### Nutritional status of under 5 children

According to the anthropometric data obtained (Table 7), 0.6% of the children investigated in Oshana region were found to be severely wasted (< 115mm). Moreover, 4.5% of the children from Oshana region were found to be moderately wasted (≥115mm and <125mm), based on MUAC cut offs. While 24.6% and 1.5% of children were severely and moderately wasted, respectively, in the Oshikoto region based on MUAC cut offs. The prevalence of acute malnutrition among children based on weight for height z-score indicated higher values (2% were severely wasted and 6.5% were moderately wasted) in the Oshikoto region compared to the Oshana region (1.1% were severely wasted (<-3 z-score) and 3.4% moderately wasted (≥ -3 and < -2 z-score)). In addition, the prevalence of underweight children based on WAZ was higher in the Oshikoto region (3.5% severely underweight (<-3 z-score) and 14.6% moderate underweight (≥ -3 and <-2 z-score)) compared to the Oshana region (0.6% severely underweight children (<-3 z-score) and 12.9% moderate underweight children (≥ -3 and <-2 z-score)) (Table 7). For the prevalence of stunting among the children based on HAZ, similar values were recorded in both regions (Oshana region: 9.0% were severely stunted (<-3 z-score) and 20.8% were moderately stunted (≥ -3 and <-2 z-score); Oshikoto region: 7% were severely stunted and 20.6% were moderately stunted). For the prevalence of overweight children based on weight for height, 4.5% and 2% of the children from Oshana and Oshikoto regions, respectively, were overweight (WHZ > 2), while 1.1% and 2% of children were respectively severely overweight (WHZ > 3) (Table 7).

**Table 7:** Prevalence of underweight, stunting and wasting in Oshana and Oshikoto regions

Category	Cut off point (Z- score)	Prevalence (%)	
		Oshana	Oshikoto
Prevalence of acute malnutrition by age, based on MUAC cut off's			
Severe wasting	(< 115 mm)	0.6%	24.6%
Moderate wasting	(MUAC ≥115 mm and < 125 mm)	4.5%	0.5%
Normal	(MUAC ≥125 mm)	94.9%	74.9%
Prevalence of acute malnutrition by age, based on weight-for-height z-scores			
Severe wasting	(WHZ<-3 z-score)	1.1%	2%
Moderate wasting	(WHZ ≥ -3 SD and WHZ<-2 SD z-score)	3.4%	6.5%
Normal	(WHZ ≥-2 SD z score)	95.5%	91.5%
Prevalence of underweight by age, based on weight-for-age z-scores			
Severe underweight	(WAZ<-3 z-score)	0.6%	3.5%
Moderate underweight	(WAZ<-2 SD z-score and WAZ ≥-3 SD z-score)	12.9%	14.6%
Normal	(WAZ≥-2 z score)	86.5%	81.9%
Prevalence of stunting by age based on height-for-age z-scores			
Moderate stunting	(HAZ<-2 SD z-score and HAZ ≥-3 SD z-score)	20.8%	20.6%
Severe stunting	(HAZ<-3 SD z-score)	9.0%	7.0%
Normal	(HAZ ≥-2 SD z score)	70.2%	72.6%
Prevalence of overweight by age, based on weight for height			
Severe overweight	(WHZ > 3)	1.1%	2.0%
Overweight	(WHZ > 2 SD)	4.5%	2.0%

Key: *WAZ* Weight for age Z-score, *HAZ* Height for age Z-score, *WHZ* Weight for height Z-score and *SD* Standard deviation

### Association of food consumption pattern with nutritional status

The Chi-square results revealed no association between minimum dietary diversity, minimum milk feeding frequency, and minimum acceptable diet and nutritional status indicators among children from both regions (*p* > 0.05). However, a significant association was found between wasting and amount of food consumed, and between beans consumption and overweight among children in the Oshana region (*p* < 0.05 and after Bonferroni correction) (Table 8). Conversely, Multinomial logistic regression indicated a strong positive association between the amount of food consumed and moderate acute malnutrition (OR of 10.514), as well as a strong positive association between beans consumption and overweight (OR of 21.250) and a strong negative association between beans consumption and severe overweight (OR of 6.994E-7). In the Oshikoto region, significant associations (*p* < 0.05) were observed between frequency of food consumption/minimum meal frequency and wasting, length of breastfeeding and overweight, consumption of pearl millet thick/thin porridge and wasting, and fresh fish consumption and wasting (Table 8). Multinomial logistic regression revealed a strong negative association between frequency of meal consumption and severe or moderate wasting (OR of 0.106 and 9.217E-9, respectively), between the length of breastfeeding and wasting (OR of 0.930), and a strong negative association between pearl millet thick/thin porridge and fresh fish with severe wasting (OR of 1.609E-7 and 2.957E-8, respectively).

**Table 8:** Cross tabulation of food consumed and anthropometric data in Oshana and Oshikoto regions using Chi-square

Variable	Regions	Underweight	Wasting (WHZ)	Wasting (MUAC)	Stunting	Overweight
Frequency of food consumption	Oshana	0.972	0.669	0.336	0.796	0.955
	Oshikoto	0.895	0.213	<0.001^**^	0.604	0.912
Amount of food consumed	Oshana	0.009	0.059	<0.001^**^	0.304	0.929
	Oshikoto	0.636	0.469	0.543	0.760	0.181
Minimum dietary diversity	Oshana	0.667	0.280	0.531	0.747	0.182
	Oshikoto	0.560	0.854	0.907	0.341	0.549
Minimum acceptable diet	Oshana	0.233	0.781	0.525	0.378	0.242
	Oshikoto	0.895	0.895	0.845	0.825	0.980
Minimum meal frequency	Oshana	0.795	0.205	0.067	0.484	0.702
	Oshikoto	0.595	0.055	<0.001**	0.615	0.623
Minimum milk feeding frequency	Oshana	0.348	0.698	0.438	0.144	0.313
	Oshikoto	0.637	0.826	0.504	0.848	0.921
Length of breastfeeding	Oshana	0.865	0.857	0.252	0.039	0.403
	Oshikoto	0.515	0.055	0.033	0.360	<0.001**
Pearl millet thick/ thin porridge	Oshana	0.809	0.787	0.759	0.707	0.815
	Oshikoto	0.888	0.025	<0.001**	0.747	0.722
Maize thick/thin porridge	Oshana	0.417	0.642	0.491	0.033	0.697
	Oshikoto	0.637	0.311	0.490	0.848	0.772
Beans	Oshana	0.797	0.939	0.931	0.733	0.005^**^
	Oshikoto	0.398	0.678	0.247	0.762	0.679
*Oshikundu*	Oshana	-	-	-	-	-
	Oshikoto	0.637	0.490	0.311	0.848	0.772
*Ombidi*	Oshana	0.582	0.260	0.842	0.674	0.170
	Oshikoto	0.835	0.494	0.548	0.718	0.153
Fresh fish	Oshana	0.495	0.824	0.800	0.658	0.296
	Oshikoto	0.047	< 0.001^**^	0.290	0.323	0.476
Chicken	Oshana	0.738	0.920	0.908	0.813	0.025
	Oshikoto	0.790	0.826	0.504	0.848	0.772
Beef	Oshana	0.537	0.842	0.821	0.056	0.232
	Oshikoto	0.506	0.529	0.548	0.878	0.404
Dried fish	Oshana	0.206	0.840	0.606	0.401	0.093
	Oshikoto	0.497	0.538	0.050	0.139	0.200
Rice	Oshana	0.090	0.206	0.136	0.178	0.540
	Oshikoto	0.461	0.806	0.060	0.687	0.037
Macaroni	Oshana	0.406	0.393	0.278	0.123	0.460
	Oshikoto	0.017	0.051	0.517	0.151	0.279
Milk	Oshana	-	-	-	-	-
	Oshikoto	0.509	0.556	0.856	0.642	0.537
Potatoes	Oshana	-	-	-	-	-
	Oshikoto	0.160	0.020	0.123	0.226	0.967

^**^Significant at *p<*0.05 and after Bonferroni correction (*p*≤ adjusted significance level) -constant

## Discussion

### Demographic characteristics of rural households

The demographic data showed that, only 5% and 4% of the childcare providers from Oshana and Oshikoto regions, respectively received tertiary education. The low level of education among them could be attributed to the cultural practice in rural areas where most women are married at an early age as well as to high rates of teenage pregnancies in rural areas which lead to early school dropouts [18]. Also, childcare providers from both regions were mostly subsistence farmers and this could be explained by the low level of education among the respondents which may be contributing to inability of securing better employment. Policy interventions focused on nutrition and childcare for childcare providers are recommended.

Six months of exclusive breast feeding is highly recommended by the World Health Organization as the breastmilk is a vital source of sustenance to children [19]. Additionally, breastmilk is known to have a significant effect on cognition, behavioural, and mental health in children [20]. Therefore, this may support the findings of this study that majority of the children were breastfed for more than 12 months and majority of them (95.8%) do not experience any health challenge as reported by their care providers. This study found a significant association between breastfeeding length and wasting in Oshikoto region. It is therefore important that children be exclusively breast-fed for the first 6 months of life as recommended by WHO and thereafter they should be fed complementary foods that are nutritionally safe and adequate while continuing to be breast-fed until 2 years and above [12]. At policy levels, it is recommended that initiatives that support and educate mothers on benefits of extended breastfeeding be implemented and strengthened.

### Food consumption pattern

A diversified diet is essential for children to meet their nutritional demands and ensure normal health, growth, and development. The dietary diversity scores in this study refers to the number of main food groups that were consumed by under 5 years children for the minimum of 5 days in a week. The mean dietary diversity scores of children from Oshana and Oshikoto regions were 4±1 SD and 5±1 SD, respectively. These figures were higher than those reported in a study conducted in Ghana [21]. The minimum dietary diversity scores among children from the Oshana and Oshikoto regions aligned with those documented in children from Sub-Saharan Africa in previous studies [14, 21–23].

A low dietary diversity score observed among the children in this study indicates that most children, primarily in Oshikoto region, are unlikely to meet their nutritional demands. This suggests the need to implement community-based programs to educate childcare providers about the importance of diversified diets and supporting initiatives that enhance agricultural practices in rural areas. For instance, establishing community gardens to ensure the availability of a variety of nutrient-rich food crops in rural areas. However, this study did not establish an association between minimum dietary diversity and nutritional status. These results are consistent with the study of [24]. Other studies in Africa have found an association between dietary diversity and nutritional status [14, 25–27].

Grains, roots and tubers and flesh foods, were popularly consumed by children from both regions, followed by legumes and nuts, as well as other fruits and vegetables. These consumption patterns are in accordance with the findings of other studies [25, 28, 29].

The high consumption of staple food among children in rural household of Oshana and Oshikoto regions may also be attributed to their availability in terms of socioeconomic status, cultural beliefs, and traditional dietary practices. Jang [30] reported that, socio economic status may affect and defines the availability of food in terms of quantity and quality as well as in relation with cultural and food practices in the households. Thompson and Amoroso [31] further reported the existence of a shift from diverse diets to the diets that are mainly high in carbohydrates in the Sub-Saharan Africa, due to declining incomes. Oniang’ o, Mutuku and Malaba [32] also reported that, in African communities, locally available staples such as maize, cassava and sweet potatoes generally form the basis of a meal, which is complemented with legumes or food from animal sources to make the meal nutritious. These findings align with this study, as staple cereals, *ombidi*, beans, chicken, beef, *oshikundu* and fish were amongst the foods that were mostly consumed by the children in a week.

The low consumption of dairy products, eggs or vitamin A-rich fruits and vegetables in this study have also been documented by previous authors [33, 34]. This is a nutritional concern among these populations, as WHO recommends frequent consumption of eggs, fruits, and vegetables for optimal growth and development [12].

The consumption of vitamin A rich fruits and vegetables among the study subjects was the least compared to other foods such as cereals. However, vegetables such as *ombidi* and beans were mostly consumed by the children from both regions, and this may be attributed to their local availability in the regions. The low consumption of variety of fruits and vegetables by the study subjects is consistent with that of Anane and co-workers [28] who found out that consumption of grains, root, and tubers were relatively higher in their study but low for Vitamin A-rich fruits and vegetables for under five years children in Ghana. The study findings also concur with that of Vahatalo and co-workers [29] who observed a high consumption of millet, maize and milk as well as an extremely low consumption of fruits and vegetables among children in Kaokoland in Namibia. Fruits form a basic part of healthy nutrition despite their low consumption pattern in the study. They provide vitamins, minerals, fibre, as well as many vital substances, which aid in preventing non-infectious diseases such as cardiovascular disease, diabetes and cancer [35]. The daily consumption of fruits and vegetables is also highly recommended by the food and nutrition guidelines for Namibians [36]. However, the low consumption of fruits and vegetables in the study may also be influenced by the availability of fruits in the region, education level and affordability [37]. However, this study could not find an association between education level and food consumption. Nevertheless, the low consumption pattern of fruits in the study may indicate unhealthy and unbalanced dietary food consumption among rural children, which may subsequently escalate susceptibility to various diseases and children mortality.

The findings of this study also revealed that, majority of the children from Oshana and Oshikoto regions were fed food that was either produced at home and from the market. These findings align with research results reported by other studies [38, 39]. The consumption pattern observed in this study might also be influenced by economic and climatic factors. Namibia being one of the African countries which are affected by drought, experiences minimal agricultural production [40]. Despite most childcare providers being subsistence farmers, they are unable to produce sufficient food for their families, leading to the necessity to supplement their staple food by purchasing items from the market.

Additionally, the study found that the proportion of children receiving solid, semi-solid, or soft foods per day differed between the two regions. Children from Oshikoto mostly consumed meals 1 to 3 times a day, while those from Oshana mostly consumed meals 4 to 6 times a day. The recommended minimum meal frequency for non-breastfeeding children is 4 or more meals a day [12]. A higher proportion of children (70%) from the Oshana region met the recommended minimum meal frequency compared to children from the Oshikoto region (32%). The minimum meal frequency for both regions was lower than those reported by Belew and co-workers [22]. Additionally, the minimum meal frequency for the Oshikoto region was lower than the 41.9% reported in Sub-Saharan Africa [41], but the recorded 41.9% in Sub-Saharan Africa was also lower than that found in the Oshana region.

The low minimal meal frequency observed in the Oshikoto region indicates that most children were not meeting their energy requirements, suggesting vulnerability to malnutrition [42]. The lower frequency of food consumption per day among some of the study participants may be attributed to limited food availability in rural households and poor income. The families might have insufficient income to purchase a variety of food for their children, leading them to be fed only a few times a day, conserving food for the next day. This is supported by Anane and co-workers [28] who revealed that, food affordability and availability may affect the consumption of food among children. The children from rich families tend to consume more food compared to those from poorer families.

Most children consumed either 375g or 250g of food per meal, and only a few had more than 7 meals a day or consumed 500g of food per meal across both regions. These patterns of meal consumption are consistent with the observations made by Williams and Smith [39].

Furthermore, a small proportion (27% and 0.5%) of the children from Oshana and Oshikoto regions respectively, were fed a minimum acceptable diet. A minimum acceptable diet is crucial to children for their growth and development, despite its low prevalence in this study. However, this study could not establish a significant association between minimum acceptable diet and nutritional status. The lack of association between minimal acceptable diet and nutritional status is in accordance with the findings of Niyigena and co-authors [24]. The low prevalence of minimum acceptable diet indicates the risk of children to malnutrition. It is therefore important that children maintain an adequate dietary intake to attain their daily energy by consuming a varied, healthy, and balanced diet. This underscores the importance of fostering collaboration with health care facilities and conducting awareness campaigns in rural areas to educate childcare providers on proper feeding practices and nutrition for children. Moreover, implementation of policies becomes imperative to improve food environments and eliminating access barriers to diversified diets, thereby mitigating high prevalence of malnutrition among rural children.

### Nutritional status

Anthropometric measurements are normally used in the assessment of nutritional status of individuals in communities. Evaluation of the nutritional status of people was established to be a crucial tool in public health and a possible indicator of living standards [43]. According to the findings of this study, the recorded prevalences of wasting, and underweight from the two regions are in line with the survey results of Fred [44] in Namibia who reported that, nearly 9% of the children were wasting and 26% were underweight. The prevalence of wasting and underweight among the children under 5 years has also been reported in previous studies in rural areas of Sub-Sahara Africa, however their figures were found higher than for this study [44–47]. However, this study may potentially overlook children who were wasting as bilateral pitting oedema was not assessed.

The prevalences of stunting recorded in this study for Oshana and Oshikoto regions are higher than the findings of the Namibian Demographic and Health Survey (NDHS) (2013) which reported that nationally, 24% of children under five years were stunted and 8% were severely stunted with a high percentage of stunting accounted for children from rural areas [4]. The stunted children’s heights were found to be lower, relative to their ages and this is an indicator of chronic malnutrition among those children.

Even though the results of the study showed that many of the children who participated in the study had normal weight for age, normal height for age and normal weight for height, the observed prevalence of wasting (24.6%), overweight (5.6%), underweight (18.1%), and stunting (29.8%) are a source of public health concern. Thus, improvement of the health and nutritional status of children through adequate dietary intake can diminish the effects of malnutrition on growth and development of rural children. Several studies have documented the factors that are responsible for stunting and wasting among the children under 5 years. These factors include inappropriate infant feeding practices, optimal duration of exclusive breastfeeding, socioeconomic status (education level, occupation, household income) as well as living in rural areas [45, 48, 49].

This study observed the associations between breastfeeding length, the amount of food consumed, frequency of food consumption/MMF and the types of food consumed by children under the age of 5 with wasting. The consumption of pearl millet thick/ thin porridge, fresh fish consumption and higher frequency of meal consumption was associated with a significant decrease in the odds of having severe/moderate wasting, suggesting a strong protection against wasting which is of practical importance. Whereas an increase in the amount of food was associated with the increase in the odds of children being moderately wasted. Conversely, longer breastfeeding duration showed a non-significant decrease in the odds of severe overweight (*p* > 0.05), suggesting insufficient evidence to conclude a significant effect on severe overweight odds.

Nevertheless, the observed associations between food consumption patterns and nutritional status in this study align with those reported by other authors [50, 51] and are consistent with several studies in Africa and Asia which indicated that, the amount of food (small portion sizes), inadequate dietary diversity and low meal frequencies contribute to poor nutritional status of children including stunting, wasting or underweight [52–55]. Underweight and wasting are forms of acute malnutrition which may results from recent food deficit, thus, a reduced frequency of meal consumption among children, which was also observed among some of the study participants may result in low energy levels escalating the risk of adverse nutritional status [53].

Additionally, staple cereals which are regularly consumed in the study may contain natural toxicants which may potentially interfere with the absorption of essential micronutrients from foods consumed by children [50]. This could result in compromised nutritional status such as wasting which was linked to the consumption of pearl millet thick/thin porridge among the study participants. Hence, there is a crucial need for a comprehensive investigation into the natural toxicants present in these staple foods. Moreover, an association was observed between beans consumption and overweight among children from Oshana region. However, it was noted that vegetables consumption appeared to be linked to a reduction in overweight cases [56]. These findings suggest that other factors including dietary feeding practices (example; the consumption of food high in saturated fat and sugar) as well as lack of physical activities might be the potential contributors to overweight among these children [57]. However, to gain a comprehensive understanding of the complex factors influencing overweight among these children, further research needs to be conducted. Promoting healthy dietary habits and addressing the underlying socio-economic factors that may influence food consumption patterns in rural areas may improve the nutritional status and overall health of children.

## Conclusion

This study revealed that children under 5 years from rural households from the Oshana and Oshikoto regions commonly consumed staple foods but had limited intake of vitamin A-rich fruits and vegetables. Both regions exhibited low minimum dietary diversity scores (55.6% in Oshana, 7% in Oshikoto). Minimum meal frequency was higher in Oshana (69.7%) compared to Oshikoto (32%), but the achievement of a minimum acceptable diet is low for both (27% in Oshana, 0.5% in Oshikoto). These findings suggest that the under 5 years children from both regions were receiving an inadequate diet, potentially failing to meet the nutritional requirements necessary for proper growth and development. The study identified prevalent wasting, underweight, stunting, and overweight, with no significant association between nutritional status and complementary feeding indicators. However, associations were found between some food types, meal frequency, breastfeeding duration, and wasting or overweight. There is a need for establishing programs to promote the improvement of food environments and eliminating access barriers to diversified diets among children in rural areas. Thus, these will address a high prevalence of malnutrition among children in rural Northern Namibia.

## Data Availability

No datasets were generated or analysed during the current study.

## Abbreviations

cm: Centimetre
ENA: Emergency Nutrition Assessment software
g: Gram
HAZ: Height-for-age Z-scores
kg: Kilograms
MUAC: Middle-upper arm circumference
mm: Millimetres
MMFF: Minimum milk feeding frequency
MDD: Minimum dietary diversity
MMF: Minimum meal frequency
MAD: Minimum acceptable diet
n: Population size
OR/(s): Odd ratio/s
SD: Standard deviations
SPSS: Statistical Package for Social Sciences
UNICEF: United Nations International Children's Emergency Fund
WAZ: Weight-for-age Z-scores
WHZ: Weight-for-height Z-scores
WHO: World Health Organization
